# Female mice may have exacerbated catabolic signalling response compared to male mice during development and progression of disuse atrophy

**DOI:** 10.1002/jcsm.12693

**Published:** 2021-03-05

**Authors:** Megan E. Rosa‐Caldwell, Seongkyun Lim, Wesley A. Haynie, Jacob L. Brown, John William Deaver, Francielly Morena Da Silva, Lisa T. Jansen, David E. Lee, Michael P. Wiggs, Tyrone A. Washington, Nicholas P. Greene

**Affiliations:** ^1^ Cachexia Research Laboratory, Exercise Science Research Center, Department of Health, Human Performance and Recreation University of Arkansas Fayetteville AR USA; ^2^ Exercise Muscle Biology Laboratory, Exercise Science Research Center, Department of Health, Human Performance and Recreation University of Arkansas Fayetteville AR USA; ^3^ Integrative Physiology and Nutrition Laboratory Name, Department of Health and Kinesiology University of Texas at Tyler Tyler TX USA; ^4^ Department of Health, Human Performance and Recreation Baylor University Waco TX USA

**Keywords:** Protein catabolism, Males, Females, Muscle loss, Protein anabolism, Sex differences

## Abstract

**Background:**

Muscle atrophy is a common pathology associated with disuse, such as prolonged bed rest or spaceflight, and is associated with detrimental health outcomes. There is emerging evidence that disuse atrophy may differentially affect males and females. Cellular mechanisms contributing to the development and progression of disuse remain elusive, particularly protein turnover cascades. The purpose of this study was to investigate the initial development and progression of disuse muscle atrophy in male and female mice using the well‐established model of hindlimb unloading (HU).

**Methods:**

One hundred C57BL/6J mice (50 male and 50 female) were hindlimb suspended for 0 (control), 24, 48, 72, or 168 h to induce disuse atrophy (10 animals per group). At designated time points, animals were euthanized, and tissues (extensor digitorum longus, gastrocnemius, and soleus for mRNA analysis, gastrocnemius and extensor digitorum longus for protein synthesis rates, and tibialis anterior for histology) were collected for analysis of protein turnover mechanisms (protein anabolism and catabolism).

**Results:**

Both males and females lost ~30% of tibialis anterior cross‐sectional area after 168 h of disuse. Males had no statistical difference in MHCIIB fibre area, whereas unloaded females had ~33% lower MHCIIB cross‐sectional area by 168 h of unloading. Both males and females had lower fractional protein synthesis rates (FSRs) within 24–48 h of HU, and females appeared to have a greater reduction compared with males within 24 h of HU (~23% lower FSRs in males vs. 40% lower FSRs in females). Males and females exhibited differential patterns and responses in multiple markers of protein anabolism, catabolism, and myogenic capacity during the development and progression of disuse atrophy. Specifically, females had greater mRNA inductions of catabolic factors *Ubc* and *Gadd45a* (~4‐fold greater content in females compared with ~2‐fold greater content in males) and greater inductions of anabolic inhibitors *Redd1* and *Deptor* with disuse across multiple muscle tissues exhibiting different fibre phenotypes.

**Conclusions:**

These results suggest that the aetiology of disuse muscle atrophy is more complicated and nuanced than previously thought, with different responses based on muscle phenotypes and between males and females, with females having greater inductions of atrophic markers early in the development of disuse atrophy.

## Introduction

During many chronic diseases, muscle wasting is a concurrent pathology and a significant predictor of mortality.^1^ Disuse‐induced muscle atrophy is a common pathology experienced by patients undergoing hospital stays such as within intensive care units (ICUs). Specifically, reduced muscle strength is associated with increased mortality and length of hospital stay^2^ as well as 30% greater healthcare costs.^3^, ^4^ Muscle wasting occurs rapidly, with significant atrophy in as little as 3–7 days.^5^ However, mechanisms contributing to this rapid progression in muscle loss are not fully elucidated, making the development of effective therapeutics difficult.

While it is clear that muscle wasting is a large mediator of quality of life, there remains little comparative information on how muscle wasting initially develops between male and female organisms. During extended bed rest, there is evidence that female humans are more likely to experience ICU‐associated muscle weakness,^6^ which may contribute to the elevated ICU mortality observed in female humans.^7^ Sex differences in clinical outcomes may be related to differences in muscle physiology between male and female organisms.^8^ Muscle size is maintained through a delicate balance of protein synthesis and degradation. During disuse atrophy, there is a commonly observed net decrease in anabolic processes [Akt/mTOR signalling, fractional protein synthesis rate (FSR), satellite cell proliferation, etc.] and net increase in catabolic processes (ubiquitin–proteasomal signalling, autophagy activation, etc.).^9^, ^10^ Recent evidence suggests that females may have lower content of moderators of ubiquitin–proteasomal degradation,^11^ but how these potential differences correspond to atrophic stimuli remains unknown.

A recent review from our group^8^ examining sex differences in muscle atrophy clearly highlighted the dearth of data comparing susceptibility and mechanisms of atrophy in between sexes. Because muscle wasting is clearly related to increased mortality,^2^ understanding differences between disuse‐induced muscle atrophy between males and females is critical for the development of therapies to treat muscle loss. Therefore, the purpose of this study was to investigate mechanisms of protein turnover during the initial development and progression of disuse muscle atrophy in male and female mice. We hypothesized that males and females would have different cellular signalling profiles contributing to muscle loss; however, this study was primarily description and we did not hypothesize which precise mechanisms would differ between sexes.

## Methods

### Animal protocol

All procedures were approved the University of Arkansas Institutional Care and Use Committee; ~100 male and female C57BL/6J mice (50 of each sex) were purchased from Jackson Laboratories (Bar Harbor, ME, USA). At 10 weeks of age, animals were divided into experimental groups of 0 (control), 24, 48, 72, or 168 h of hindlimb unloading (HU), corresponding to 1, 2, 3, or 7 days. Animals were hindlimb unloaded to induce disuse atrophy as previously described.^12^ A more through description of the HU protocol can be found in Supporting Information, *Data* S1. At designated time points, animals were anaesthetized using 2% isoflurane, with care taken to avoid reloading of hindlimbs. Hindlimb tissues were collected and snap frozen in liquid nitrogen for further analysis, and animals were euthanized while under anaesthesia. During HU, food was dampened with tap water to facilitate consumption; however, this negated our capacity to reliably measure food consumption. Visual inspection of the food indicated that all animals consumed food during the designated interventions.

### Histological analysis

Immunofluorescent staining was completed as described with minor modifications.^13^ At tissue harvest, tibialis anterior (TA) muscles were frozen in optimal cutting temperature compound (OCT compound). Tissues were then cut to 10‐μm‐thick cross sections using a Leica CM1859 cryostat (Leica Biosystems, Buffalo Grove, IL, USA). Slides were stained using immunofluorescent antibodies for analysis of cross‐sectional area (CSA) across fibre types.^14^ During staining, slides were maintained in dark environments to avoid photobleaching of samples. A full histological protocol can be found in *Data* S1; ~100 fibres matching the approximate proportion of different fibre types within the sample were measured. To calculate per cent distribution of each fibre, the number of a fibre type (MHCI, MHCIIA, MHCIIB, or MHCIIX/D) was divided by total number of fibres counted and multiplied by 100. Because of lack of MHCI fibres (<1 fibre per animal in the TA), data were not analysed for MHCI.

### Fractional protein synthesis rates

A full description of the protocol can be found in *Data* S1. FSRs were quantified as described previously.^15^ Twenty‐four hours prior to tissue harvest, all animals received 20 μL/g body mass of 99.9% D_2_O (Millipore Sigma, 151882‐1L, Poole, UK) via intraperitoneal injection to achieve an ~2% enrichment of the total body water with deuterium. Following intraperitoneal injections of D_2_O, cage water was supplemented with 4% deuterium to ensure maintenance of body water enrichment.^16^ Animals in the 24 h of disuse group were injected with D_2_O and then immediately hindlimb unloaded. FSR was assessed in TA and gastrocnemius muscles to assess fast‐twitch and mixed‐fibre types, respectively.

### Immunoblotting

Immunoblotting to measure signalling pathways related to protein synthesis and degradation was measured as we previously described.^17^ A full description of the immunoblotting protocol and antibodies used is found in *Data* S1. Similar to mRNA analysis, extensor digitorum longus (EDL) and gastrocnemius muscles were analysed to investigate differential responses across different muscles. The soleus could not be analysed for immunoblotting due to limitations in amount of sample.

### Real‐time PCR

Samples were homogenized and mRNA isolated as we have previously described^17^ using a commercial kit (Thermo Fisher, PureLink™ RNA Mini Kit, Cat#12183025). A more complete description of protocol and probes used is found in *Data* S1. mRNA was assessed in EDL, gastrocnemius, and soleus muscles to represent fast‐twitch, mixed‐fibre, and slow‐twitch muscle groups, respectively.

### Statistics

Within each sex, outcome variables were measured with one‐way analysis of variance with independent factors of time unloading, and Tukey's *post hoc* test was used to determine differences between means. All outcome variables were analysed through trend analysis as we previously described.^18^ This analysis was used to determine fluctuations in outcome variables that may not reach statistical significance at the pairwise level but appear to influence the outcome variable. Data were analysed for linear, quadratic, and cubic trends. A linear trend demonstrated progressively greater or less content of a particular outcome variable with greater durations of HU. A quadratic trend represented a parabola‐based pattern across the outcome variable, typically coincided with a peak or trough in the data. A cubic trend represented data that appeared had two points of inflection. Significance for all analyses was denoted at *P* < 0.05. All data were analysed with SAS statistical software (SAS Institute, Cary, NC, United States).

## Results

An expanded description of results can be found in *Data* S2.

### Lower body and tissue weights following hindlimb unloading in male and female mice

Tissue weights are presented in *Table* 1. Overall, male and female animals had lower body weight and muscle weights with HU. Of note, lower muscle weights were not significant between 0 and 24 h in male mice; however, in female mice, there were statistically significant lower muscle weights within 24 h of unloading. Graphical representation of per cent losses and muscle losses normalized to body weight at time of HU is presented in *Figure* S1.

**Table 1:** Raw body weights and tissue weights from animals in the present study

		0 h	24 h	48 h	72 h	168 h
Body weight (g)	Males^a^	23.10 ± 0.52^a^	22.12 ± 0.23^a^ ^,^ ^b^	21.18 ± 0.33^b^	21.89 ± 0.46^a^ ^,^ ^b^	21.23 ± 0.45^b^
	Females^b^	20.00 ± 0.04^a^	18.37 ± 0.37^a^ ^,^ ^b^	18.97 ± 0.51^a^ ^,^ ^b^	17.60 ± 0.60^b^	18.49 ± 0.22^a^
Gastrocnemius (mg)	Males^a^	117.92 ± 3.93^a^	119.68 ± 2.2^a^ ^,^ ^b^	103.81 ± 2.14^b^	108.24 ± 2.39^a^ ^,^ ^b^	99.37 ± 2.41^b^
	Females^a^	91.19 ± 1.88^a^	85.14 ± 1.53^a^	82.94 ± 1.65^b^	76.81 ± 1.92^b^	75.80 ± 1.54^b^
Soleus (mg)	Males^a^	8.31 ± 0.36^a^	8.67 ± 0.38^a^	7.15 ± 0.32^b^	6.68 ± 0.17^b^	5.10 ± 0.32^c^
	Females^a^	7.49 ± 0.26^a^	6.67 ± 0.19^b^	6.15 ± 0.71^b^	5.69 ± 0.31^b^ ^,^ ^c^	4.96 ± 0.27^c^
Plantaris (mg)	Males^a^	16.24 ± 0.41^a^	17.03 ± 0.68^a^	14.43 ± 0.33^b^	14.41 ± 0.54^b^	13.32 ± 0.31^b^
	Females^a^	13.13 ± 0.63^a^	11.06 ± 0.24^b^	10.96 ± 0.34^b^	9.84 ± 0.49^b^	10.47 ± 0.23^b^
Tibialis anterior (mg)	Males^a^	41.43 ± 1.17^a^ ^,^ ^b^	44.46 ± 0.84^b^	40.03 ± 0.59^a^	40.03 ± 0.59^a^	35.88 ± 1.06^b^
	Females^a^	33.59 ± 0.86^a^	30.74 ± 0.73^a^ ^,^ ^b^	30.52 ± 0.82^b^	28.66 ± 0.67^b^	29.62 ± 1.05^b^
Extensor digitorum longus (mg)	Males	9.66 ± 0.47	9.87 ± 0.36	9.33 ± 0.15	9.14 ± 0.34	8.95 ± 0.26
	Females	7.72 ± 0.45	7.46 ± 0.13	7.03 ± 0.31	6.57 ± 0.34	7.19 ± 0.51
Triceps (mg)	Males	95.34 ± 2.10	93.74 ± 1.71	90.82 ± 2.01	97.45 ± 2.59	89.50 ± 2.41
	Females^c^	78.130 ± 2.53^a^	77.91 ± 1.12^a^	72.19 ± 2.27^a^ ^,^ ^b^	64.83 ± 2.55^b^	72.77 ± 1.59^a^ ^,^ ^b^

Different letters represent statistical differences within that sex at Tukey‐adjusted *P* ≤ 0.05.

^a^ Linear trend within a sex.

^b^ Quadratic trend within a sex.

^c^ Cubic trend within a sex.

**Figure 1:**
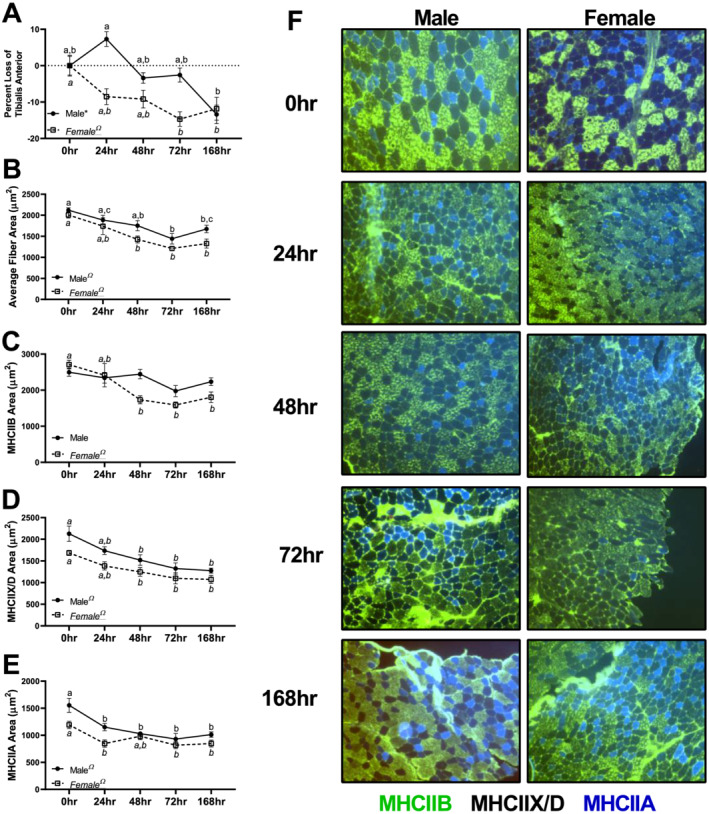
Mean fibre cross‐sectional area (CSA) data in males and females across different fibre types. *(A)* Per cent mass loss in the tibialis anterior across different durations of unloading in males and females. *(B)* Mean CSA of all fibre types combined in the tibialis anterior across different durations of unloading in males and females. *(C)* Mean CSA of MHCIIB fibres across different durations of unloading in males and females. *(D)* Mean CSA of MHCIIX/D fibres across different durations of unloading in males and females. *(E)* Mean CSA of MHCIIA fibres across different durations of unloading in males and females. *(F)* Representative images of muscle CSA data. All images were acquired at ×10 magnification. Different letters represent statistical differences at Tukey‐adjusted *P* ≤ 0.05. *Linear trend within a sex. ^Ω^Quadratic trend within a sex. ^#^Cubic trend within a sex. Female data are italicized and underlined.

Additionally, in the TA muscle, females appeared to have greater reductions in TA mass compared with males (*Figure* 1A). Specifically, males had a linear trend in per cent loss in TA mass (*P* < 0.0001, *Figure* 1A), which reached statistical significance in 168 h animals (~13% lower). In females, a quadratic trend was noted in TA per cent loss (*P* = 0.002) (*Figure* 1A). Males had significant linear or quadratic trends corresponding to lower CSA in MHCIIA and MHCIIX/D, but not in MHCIIB. However, in females, there were significant linear and quadratic trends corresponding to lower CSA across all fibre types. Frequency distribution for total fibres as well as individual fibre types can be found in *Figure* S2. Fibre composition for each fibre can be found in *Figure* S3.

### Hindlimb unloading resulted in altered fractional protein synthesis rates that differed by tissue and sex

**Figure 2:**
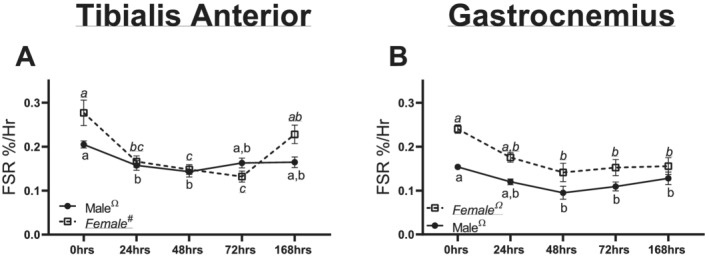
Muscle fractional protein synthesis rates (FSRs) in males and females across different durations of unloading. *(A)* Mixed FSR in the tibialis anterior of males and females across different durations of unloading. *(B)* Mixed FSR in the gastrocnemius of males and females across different durations of unloading. Different letters represent statistical differences at Tukey‐adjusted *P* ≤ 0.05. *Linear trend within a sex. ^Ω^Quadratic trend within a sex. ^#^Cubic trend within a sex. Female data are italicized and underlined.

Within the TA muscle, males had a quadratic trend noted for mixed muscle FSR (*P* = 0.0037, *Figure* 2A). In females, a quadratic trend was also observed between duration of unloading and FSR (*P* < 0.0001, *Figure* 2A). In male gastrocnemius muscle, a significant quadratic trend was observed in FSR (*P* = 0.001, *Figure* 2B). In female gastrocnemius muscle, a significant quadratic trend was also observed in FSR (*P* < 0.001, *Figure* 2B).

### Male and female mice appeared to have differential responses in phosphorylation of proteins related to protein synthesis

**Figure 3:**
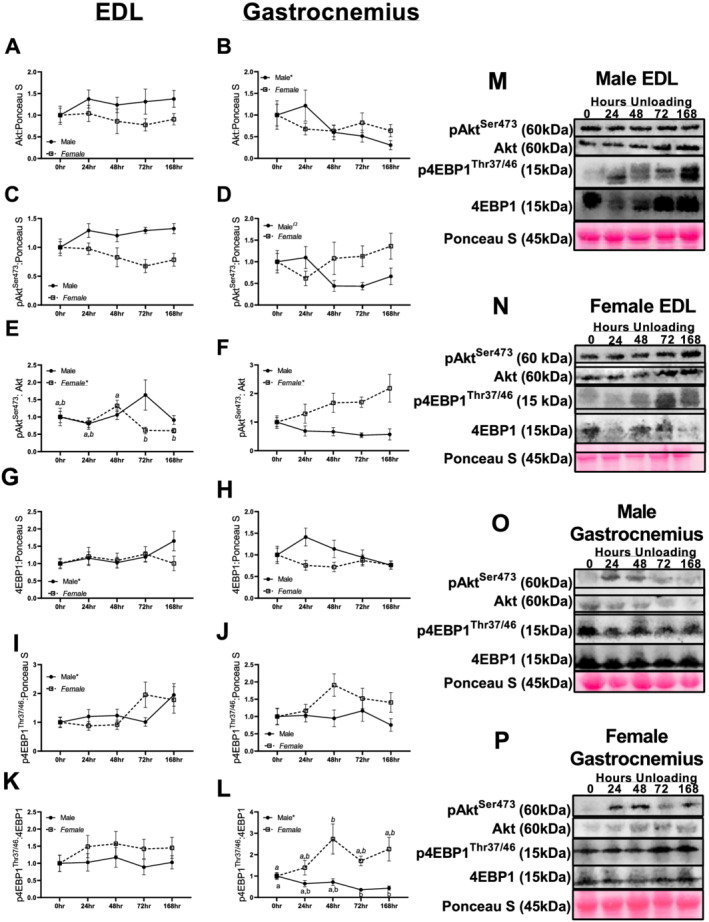
Immunoblot data for marks of protein synthesis in males and females across different durations of unloading. *(A)* Akt protein content in the extensor digitorum longus (EDL) of males and females across different durations of unloading. *(B)* Akt protein content in the gastrocnemius of males and females across different durations of unloading. *(C)* pAkt^Ser473^ protein content in the EDL of males and females across different durations of unloading. *(D)* pAkt^Ser473^ protein content in the gastrocnemius of males and females across different durations of unloading. *(E)* pAkt^Ser473^:Akt protein ratio in the EDL of males and females across different durations of unloading. *(F)* pAkt^Ser473^:Akt protein ratio in the gastrocnemius of males and females across different durations of unloading. *(G)* 4EBP1 protein content in the EDL of males and females across different durations of unloading. *(H)* 4EBP1 protein content in the gastrocnemius of males and females across different durations of unloading. *(I)* p4EBP1^Thr37/46^ protein content in the EDL of males and females across different durations of unloading. *(J)* p4EBP1^Thr37/46^ protein content in the gastrocnemius of males and females across different durations of unloading. *(K)* p4EBP1^Thr37/46^:4EBP1 protein ratio content in the EDL of males and females across different durations of unloading. *(L)* p4EBP1^Thr37/46^:4EBP1 protein ratio content in the gastrocnemius of males and females across different durations of unloading. *(M)* Representative images for EDL immunoblot data in males. *(N)* Representative images for EDL immunoblot data in females. *(O)* Representative images for gastrocnemius immunoblot data in males. *(P)* Representative images for gastrocnemius immunoblot data in females. Different letters represent statistical differences at Tukey‐adjusted *P* ≤ 0.05. *Linear trend within a sex. ^Ω^Quadratic trend within a sex. ^#^Cubic trend within a sex. Female data are italicized and underlined.

In male and female EDL muscle, there were no significant differences or trends noted in Akt content (*P* = 0.733 and *P* = 0.861, respectively, *Figure* 3A, 3M, and 3N). In male gastrocnemius, a significant linear trend was noted (*P* = 0.031, *Figure* 3B and 3N). Contrastingly, in female gastrocnemius muscle, no significant trends or pairwise differences were noted in Akt content (*P* = 0.568, *Figure* 3B and 3P). Neither males nor females had any significant differences or trends noted in pAkt^Ser473^ content in the EDL (*P* = 0.222 and *P* = 0.193, respectively, *Figure* 3C, 3M, and 3N). In male gastrocnemius muscle, a significant quadratic trend was noted in pAkt^Ser473^ content (*P* = 0.023, *Figure* 3D and 3O). Females had no significant trends between unloading conditions on pAkt^Ser473^ content in the gastrocnemius muscle (*P* = 0.521, *Figure* 3D and 3O). In male EDL muscle, there were no significant differences in pAkt/Akt ^Ser473^ ratio (*P* = 0.213, *Figure* 3E and 3M). However, in female muscle, a linear trend was noted in pAkt ^Ser473^/Akt ratios (*P* = 0.026, *Figure* 3E and 3N). In male gastrocnemius muscle, there were no statistical differences noted in pAkt^Ser473^/Akt ratio (*P* = 0.360, *Figure* 3F and 3O). In female gastrocnemius muscle, a significant linear trend was noted in pAkt^Ser473^/Akt ratio (*P* = 0.013, *Figure* 3F and 3P).

In male EDL muscle, a linear trend was noted in male 4EBP1 content (*P* = 0.011), with longer durations of unloading associated with gradually greater 4EBP1 content (*Figure* 3G and 3M). In female EDL muscle, no significant trends were detected in 4EBP1 content (*P* = 0.862, *Figure* 3G and 3N). There were no significant differences in 4EBP1 protein content in male or female gastrocnemius muscle (*P* = 0.13 and *P* = 0.47, respectively, *Figure* 3H, 3O, and 3P). In male EDL muscle, a significant linear trend was noted in p4EBP1^Thr37/45^ content (*P* = 0.015, *Figure* 3I and 3M). Contrastingly, in female EDL muscle, p4EBP1^Thr37/45^ content was not significant (*P* = 0.104). Neither males nor females had significant alterations in p4EBP1^Thr37/45^ in the gastrocnemius muscle across any unloading groups (*P* = 0.805 and *P* = 0.156, respectively, *Figure* 3J, 3O, and 3P). Neither male nor female EDL muscle had any significant differences in p4EBP1^Thr37/45^/4EBP1 content (*P* = 0.955 and *P* = 0.715, respectively, *Figure* 3K, 3M, and 3N). However, gastrocnemius males did have a significant linear trend noted in p4EBP1^Thr37/45^/4EBP1 ratio (*P* = 0.006, *Figure* 3L and 3O). Contrastingly, in female gastrocnemius, no significant trends were detected in p4EBP1^Thr37/45^/4EBP1 ratios (*P* = 0.10–0.18).

### Male and female mice exhibited divergent responses in moderators of protein anabolism

**Figure 4:**
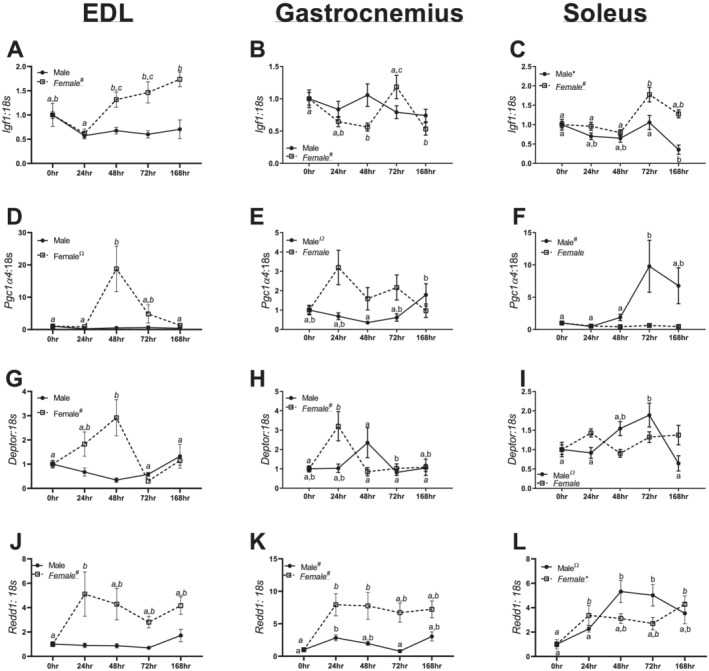
mRNA data for positive and negative moderators of protein anabolism in the extensor digitorum longus (EDL), gastrocnemius, and soleus of males and females across different durations of unloading. *(A)* *Igf1* mRNA data in males and females in the EDL. *(B)* *Igf1* mRNA data in males and females in the gastrocnemius. *(C)* *Igf1* mRNA data in males and females in the soleus. *(D)* *Pgc1α4* mRNA data in males and females in the EDL. *(E)* *Pgc1α4* mRNA data in males and females in the gastrocnemius. *(F)* *Pgc1α4* mRNA data in males and females in the soleus. *(G)* *Deptor* mRNA data in males and females in the EDL. *(H)* *Deptor* mRNA data in males and females in the gastrocnemius. *(I)* *Deptor* mRNA data in males and females in the soleus. *(J)* *Redd1* mRNA data in males and females in the EDL. *(K)* *Redd1* mRNA data in males and females in the gastrocnemius. *(L)* *Redd1* mRNA data in males and females in the soleus. Different letters represent statistical differences at Tukey‐adjusted *P* ≤ 0.05. *Linear trend within a sex. ^Ω^Quadratic trend within a sex. ^#^Cubic trend within a sex. Female data are italicized and underlined.

Within male EDL muscle, there were no significant differences noted in *Igf1* mRNA content (*P* = 0.238, *Figure* 4A). However, in female EDL muscle, a significant cubic trend was noted in *Igf1* mRNA content (*P* = 0.047, *Figure* 4A). In male gastrocnemius muscle, there were no significant differences or trends noted in *Igf1* mRNA content (*P* = 0.241, *Figure* 4B). Contrastingly, in females, a cubic trend was noted in *Igf1* mRNA content in the gastrocnemius (*P* = 0.0001, *Figure* 4B). In male soleus muscle, a cubic trend was noted in *Igf1* mRNA content (*P* = 0.005, *Figure* 4C), and a quadratic trend was noted in *Igf1* mRNA content in females (*P* = 0.0002, *Figure* 4C).

**Figure 5:**
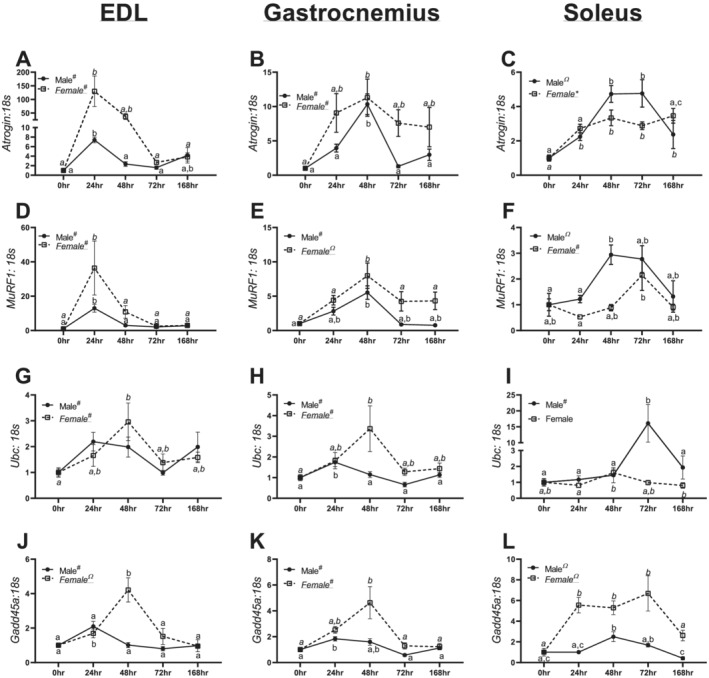
mRNA data for moderators of protein catabolism in the extensor digitorum longus (EDL), gastrocnemius, and soleus of males and females across different durations of unloading. *(A)* Atrogin mRNA content in males and females in the EDL. *(B)* Atrogin mRNA content in males and females in the gastrocnemius. *(C)* Atrogin mRNA content in males and females in the soleus. *(D)* MuRF1 mRNA content in males and females in the EDL. *(E)* MuRF1 mRNA content in males and females in the gastrocnemius. *(F)* MuRF1 mRNA content in males and females in the soleus. *(G)* Ubc mRNA content in males and females in the EDL. *(H)* Ubc mRNA content in males and females in the gastrocnemius. *(I)* Ubc mRNA content in males and females in the soleus. *(J)* Gadd45a mRNA content in males and females in the EDL. *(K)* Gadd45a mRNA content in males and females in the gastrocnemius. *(L)* Gadd45a mRNA content in males and females in the soleus. Different letters represent statistical differences at Tukey‐adjusted *P* ≤ 0.05. *Linear trend within a sex. ^Ω^Quadratic trend within a sex. ^#^Cubic trend within a sex. Female data are italicized and underlined.

In male EDL muscle, there were no differences in *Pgc1α4* mRNA content (*P* = 0.732, *Figure* 4D). Contrastingly, there was a significant quadratic trend noted in *Pgc1α4* mRNA content in female EDL muscle (*P* = 0.014, *Figure* 4D). In male gastrocnemius muscle, a significant quadratic trend was noted in *Pgc1α4* mRNA content (*P* = 0.027, *Figure* 4E), although there were no significant trends noted in *Pgc1α4* mRNA content in females (*P* = 0.084, *Figure* 5H). In male soleus muscle, a significant cubic trend was noted in *Pgc1α4* mRNA content (*P* = 0.021, *Figure* 4F); however, there were no differences in female soleus (*P* = 0.083, *Figure* 4F).

In male EDL muscle, no significant differences were noted in *Deptor* mRNA content (*P* = 0.073, *Figure* 4G). However, a cubic trend was found in *Deptor* mRNA content in females (*P* = 0.0008, *Figure* 4G). In male gastrocnemius muscle, *Deptor* content had no statistical differences (*P* = 0.13, *Figure* 4H). Contrastingly, in female gastrocnemius muscle, there was a significant cubic trend noted in *Deptor* content (*P* = 0.003, *Figure* 4H). In male soleus muscle, a significant quadratic trend was noted in *Deptor* mRNA content (*P* = 0.0004, *Figure* 4I); however, there were no differences in female *Deptor* content (*P* = 0.076, *Figure* 4I).

Within male EDL muscle, there were no significant trends in *Redd1* mRNA content (*P* = 0.157, *Figure* 4J), although females demonstrated a cubic trend in *Redd1* mRNA content (*P* = 0.007, *Figure* 4J). In male and female gastrocnemius muscle, a cubic trend was observed in *Redd1* mRNA content (*P* = 0.002 and *P* = 0.022, *Figure* 4K). Within male soleus muscle, a quadratic trend was noted in *Redd1* mRNA content (*P* = 0.0001, *Figure* 4L). However, in female soleus muscle, a linear trend was noted (*P* = 0.004, *Figure* 4L).

### RNA content of the protein ubiquitin–proteasome system showed marked inductions with unloading

In male and female EDL muscle, a cubic trend was noted in *Atrogin* mRNA content (*P* < 0.0001 and *P* = 0.0005, *Figure* 5A). In male and female gastrocnemius muscle, a significant quadratic trend was noted in *Atrogin* mRNA content (*P* = 0.0008 and *P* = 0.019, *Figure* 5B). A significant quadratic trend was noted in *Atrogin* mRNA content in male soleus muscle (*P* < 0.0001, *Figure* 5C). Contrastingly, in females, a significant linear trend was noted in *Atrogin* mRNA content (*P* = 0.0005, *Figure* 5C).

In male and female EDL muscle, a cubic trend was found in *MuRF*1 mRNA content (*P* < 0.0001 and *P* < 0.0001, *Figure* 5D). In the gastrocnemius muscle, a cubic trend was noted in *MuRF1* mRNA content in males (*P* < 0.0001, *Figure* 5E). In female gastrocnemius muscle, a quadratic trend was found in *MuRF1* mRNA content (*P* = 0.009, *Figure* 5E). In male soleus muscle, a quadratic trend was noted in *MuRF1* mRNA content (*P* = 0.0005, *Figure* 5F), whereas females had a significant cubic trend (*P* = 0.005, *Figure* 5F).

In male EDL muscle, a cubic trend was found in *Ubc* mRNA content (*P* = 0.0003, *Figure* 5G). In female EDL muscle, a pairwise difference between 0 and 48 h animals was detected with 48 h animals having ~2‐fold greater *Ubc* mRNA compared with 0 h animals (*Figure* 5G). In male and female gastrocnemius muscle, cubic trends were observed in *Ubc* mRNA content (*P* < 0.0001 and *P* = 0.033, *Figure* 5H). In male soleus muscle, a significant cubic trend (*P* = 0.002) was noted in *Ubc* mRNA content (*Figure* 5I); however, there were no significant differences in females (*P* = 0.086, *Figure* 5I).

In male EDL muscle, a cubic trend was found in *Gadd45a* mRNA content (*P* = 0.001, *Figure* 5J). However, in female EDL muscle, a significant quadratic trend was noted in *Gadd45a* mRNA content (*P* = 0.0003, *Figure* 5J). In male and female gastrocnemius muscle, significant cubic trends were observed in *Gadd45a* mRNA content (*P* < 0.0001, *Figure* 5K). Within male and female soleus muscle, quadratic trends in *Gadd45a* mRNA content were observed (*P* = 0.0002 and *P* < 0.0001, *Figure* 5L).

### Myogenesis and differentiation were differentially regulated in male and female mice

**Figure 6:**
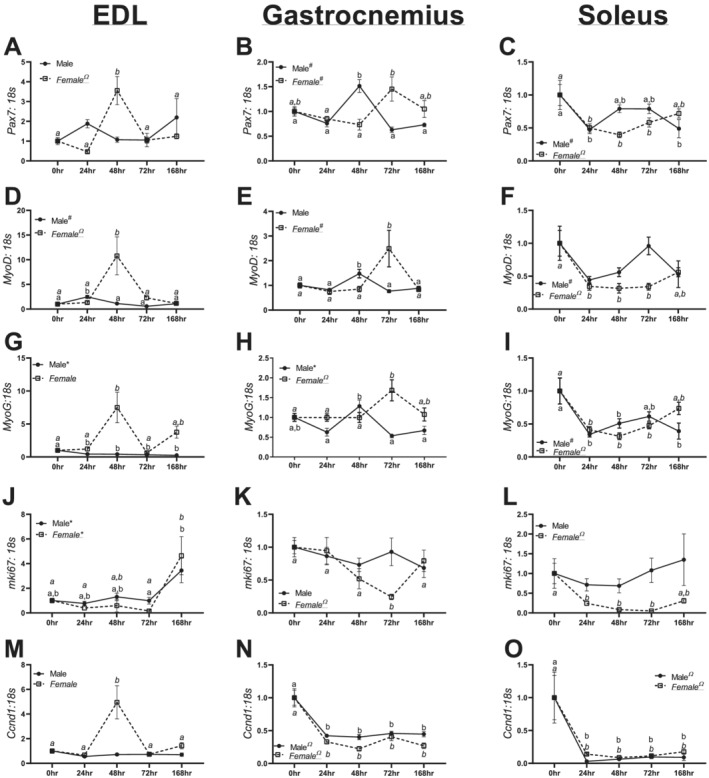
mRNA for moderators of muscle regeneration and cell cycle in the extensor digitorum longus (EDL), gastrocnemius, and soleus of males and females across different durations of unloading. *(A)* Pax7 mRNA content in males and females in the EDL. *(B)* Pax7 mRNA content in males and females in the gastrocnemius. *(C)* Pax7 mRNA content in males and females in the soleus. *(D)* MyoD mRNA content in males and females in the EDL. *(E)* MyoD mRNA content in males and females in the gastrocnemius. *(F)* MyoD mRNA content in males and females in the soleus. *(G)* MyoG mRNA content in males and females in the EDL. *(H)* MyoG mRNA content in males and females in the gastrocnemius. *(I)* MyoG mRNA content in males and females in the soleus. *(J)* mik67 mRNA content in males and females in the EDL. *(K)* mik67 mRNA content in males and females in the gastrocnemius. *(L)* mik67 mRNA content in males and females in the soleus. *(M)* Ccnd1 mRNA content in males and females in the EDL. *(N)* Ccnd1 mRNA content in males and females in the gastrocnemius. *(O)* Ccnd1 mRNA content in males and females in the soleus. Different letters represent statistical differences at Tukey‐adjusted *P* ≤ 0.05. *Linear trend within a sex. ^Ω^Quadratic trend within a sex. ^#^Cubic trend within a sex. Female data are italicized and underlined.

Within male EDL muscle, there were no significant differences noted in *Pax7* mRNA content (*P* = 0.322, *Figure* 6A), whereas a significant quadratic trend was noted in females (*P* = 0.005, *Figure* 6A). In male and female gastrocnemius muscle, cubic trends were noted in *Pax7* mRNA content (*P* = 0.008 and *P* = 0.004, *Figure* 6B). In male soleus muscle, a significant cubic trend was noted in *Pax7* mRNA content (*P* = 0.014, *Figure* 6C), although a significant quadratic trend was demonstrated in females (*P* < 0.0001, *Figure* 6C).

In male EDL muscle, a significant cubic trend was noted in *MyoD* mRNA content (*P* < 0.0001, *Figure* 6D). In female EDL muscle, a significant quadratic trend was noted in *MyoD* mRNA content (*P* = 0.019, *Figure* 6D). Within male gastrocnemius muscle, 48 h animals had ~50% lower *MyoD* mRNA content compared with 0, 24, 72, and 168 h animals (*Figure* 6E, *P* < 0.0001). In female gastrocnemius muscle, a significant cubic trend was noted in *MyoD* content (*Figure* 6E). In male soleus muscle, a cubic trend was noted in *MyoD* mRNA content (*P* = 0.0012, *Figure* 6F), whereas in females, a significant quadratic trend was noted (*P* = 0.0002, *Figure* 6F).

In male EDL muscle, there was a significant linear trend in *MyoG* mRNA content (*P* = 0.0005, *Figure* 6G); however, in female EDL muscle, a cubic trend was noted (*P* = 0.052, *Figure* 6G). In male gastrocnemius muscle, 48 h animals had ~60% greater *MyoG* mRNA content compared with 24, 72, and 168 h (*P* < 0.0001, *Figure* 6H). In female gastrocnemius muscle, a significant cubic trend was noted in *MyoG* mRNA content (*P* = 0.016, *Figure* 6H). In male soleus muscle, a significant cubic trend was noted in *MyoG* mRNA content (*P* = 0.0013, *Figure* 6I); however, a significant quadratic trend was noted in females (*P* < 0.0001, *Figure* 6I).

In male and EDL muscle, linear trends were found in *Mki67* mRNA content (*P* = 0.003 and *P* = 0.0009, *Figure* 6J). In male gastrocnemius muscle, no significant trends were noted in *Mki67* mRNA content (*P* = 0.514, *Figure* 6K); contrastingly, significant quadratic trend was found in females (*P* = 0.0008, *Figure* 6K). In male soleus muscle, there were no significant differences noted in *Mki67* mRNA content (*P* = 0.673, *Figure* 6L). However, in female soleus muscle, a significant quadratic trend was found (*P* = 0.0005, *Figure* 6L).

In male EDL muscle, there were no significant trends noted in *Ccnd1* mRNA content (*P* = 0.213, *Figure* 6M). Yet, in female EDL muscle, 48 h females had ~4‐fold greater *Ccnd1* mRNA content compared with 0, 24, 72, and 168 h animals (*P* = 0.0001, *Figure* 6M). In male and female gastrocnemius muscle, significant quadratic trends were noted in *Ccnd1* mRNA content (*P* < 0.0001 and *P* < 0.0001, *Figure* 6N). Within male and female soleus muscle, significant quadratic trends were noted in *Ccnd1* mRNA content (*P* = 0.0037 and *P* = 0.001, *Figure* 6O).

## Discussion

In the present investigation, we evaluated the time course of development and progression of disuse‐induced atrophy between biological sexes across multiple muscles. Across multiple muscle types, we find significant alterations to cellular signalling. More so, we find that female mice appear to have greater inductions of these atrophy‐related genes and greater muscle loss within the first 24–72 h of disuse. Taken together, these results suggest that 24 h of unloading is sufficient to alter many aspects of protein turnover, and these aberrations exhibit specificity depending on sex and muscle fibre phenotype.

As expected, both male and female mice exhibited progressive loss of muscle CSA. Yet female mice appeared to have greater muscle wasting after 168 h of unloading compared with males (~33.8% vs. ~21.0% lower CSAs in 168 h compared with 0 h females and males, respectively). This effect did not appear driven by relative proportions of fibres, as we did not find any dramatic differences in the proportion of MHCIIA fibres between sexes (*Figure* S3). These findings imply that female mice may have exacerbated muscle wasting compared with male mice. While speculative, this finding would support clinical data suggesting that female humans are at greater risk of ICU‐associated muscle weakness and subsequent mortality.^6^, ^7^ Moreover, in male mice, MHCIIB fibres maintained fibre area, while in female mice, there was a 34% reduction in CSA of these fibres. Yet in MHCIIA fibres, male mice appeared to have proportionately more CSA loss (~34% difference between 0 and 168 h) compared with female mice (28% difference between 0 and 168 h) potentially suggesting subtle sex differences in fibre‐type susceptibility to disuse‐induced atrophy. Prior works in cancer cachexia models have noted fibre‐type susceptibility to wasting may be different between sexes^19^, ^20^ although these studies included only male^19^ or female^20^ mice and did not include both sexes. Taken together, our data combined with prior literature implies different fibre‐type susceptibly to atrophy between males and females, with males preferentially protecting MHCIIB fibres in disuse. Regardless, while sex differences in degree of muscle loss across fibre types may exist, disuse clearly resulted in reduced muscle CSA. Considering differential effects on muscle fibre types, we performed downstream analyses on muscles with classically fast‐twitch, mixed, and slow‐twitch phenotypes to delineate differences in anabolic and catabolic signalling.

To examine the effects of disuse on muscle protein anabolism across time, we utilized deuterium oxide labelling methods in the predominantly fast‐twitch TA and mixed‐fibre‐type gastrocnemius. Similar to prior works in HU, we find a reduction in protein synthesis.^21^ More so, this drop in FSR occurs quickly, with only 24 or 48 h of disuse sufficient to result in lowered FSRs in the TA and gastrocnemius, respectively. Similar findings of lowered FSRs have been well documented in human research, with 5–21 days of immobilization or bed rest, sufficient to lower protein synthetic rates 30–48%.^22^, ^23^ In the current study, female mice exhibited greater losses to FSR in response to HU within the first 48 h. Across both the TA and gastrocnemius, female mice had greater losses in FSR compared with male mice within 48 h (~23% lower FSR in males vs. ~40% lower in females in the TA and ~29% lower in males vs. ~36% lower in females). These data also correspond to the apparent greater loss in muscle fibre CSA and mass we noted in the female TA (*Figure* 1). Notably, prior works investigating protein synthesis after disuse atrophy were conducted almost exclusively in male subjects.^21^, ^22^, ^23^, ^24^, ^25^, ^26^, ^27^ Taken together, these data imply that in the early phases of disuse, female mice have greater decrements to protein synthesis compared with male mice, which corresponds alterations in muscle size and CSA. It is also notable that between control male and female mice, females appear to have greater protein synthetic rates at baseline, which warrants further investigation on the clinical relevancy of this finding.

Interestingly, despite reduced protein synthetic function, we did not observe robust alterations to classic insulin–mTORC1 phosphorylation signalling. Rather, a further examination of mTORC1 repressors *Redd1* and *Deptor* demonstrated dramatic inductions of these genes early in the development of HU. *Deptor* and *Redd1* inhibit mTORC1 activity, effectively limiting protein synthesis.^28^, ^29^ Female mice experienced large inductions of these genes across the EDL, gastrocnemius, and soleus muscles, often within 24 h of disuse. The greater induction of *Deptor* and *Redd1* in females, particularly in the fast EDL, complements our FSR data in the fast‐twitch TA muscle and enhanced susceptibility of MHCIIB fibres to atrophy and further implies that female mice are more responsive to the initiation of disuse atrophy compared with male mice, and this responsiveness is augmented in fast and mixed‐fibre muscles.

Similar to prior works, we find robust inductions in mRNA content to moderators of the protein ubiquitin catabolic system, including *Atrogin*, *MuRF1*, *Ubc*, and *Gadd45a*.^30^, ^31^, ^32^ As the EDL muscle is highly glycolytic and typically less susceptible to disuse atrophies, it is notable that despite robust inductions of catabolic markers in both male and female mice, the EDL did not undergo significant muscle wasting by wet mass. This finding complements our prior works where overexpression of PGC‐1α was sufficient to blunt induction of *Atrogin* and *MuRF1*, but not protect against disuse‐induced muscle loss.^12^ These two findings highlight the necessity of combining phenotypic and signalling data in order to reach robust conclusions on muscle pathologies. Perhaps most notably, female mice, across multiple fibre types and markers of muscle catabolism, appear to have exacerbated inductions of protein catabolism markers compared with male mice. For example, female mice had visually greater inductions of *Atrogin*, *MuRF1*, *Ubc*, and *Gadd45a* in the EDL and gastrocnemius between 24 and 48 h of disuse (*Figure* 5). Large inductions in atrogenes occurred in the EDL and gastrocnemius, composed of more fast‐twitch/mixed muscle fibre types. Correspondingly, we noted possible sex differences in CSA, which more strongly influenced MHCIIB and MHCIIX/D within females (*Figure* 1). Taken together, our data suggest potential sex differences that appear to be more prominent in fast‐twitch/mixed muscles.

Finally, we examined mechanisms of skeletal muscle regeneration. We find significant aberrations to moderators of satellite cell activation and proliferation, with non‐linear (peaks and valleys) alterations in the EDL and gastrocnemius muscle and reduced *Pax7*, *MyoD*, and *MyoG* mRNA content in the soleus muscle in both male and female mice. In both human and murine models of disuse, satellite cell dynamics are altered.^33^, ^34^ Prior works have found greater satellite cell proliferation in the very early development of disuse atrophy in the gastrocnemius muscle (6 h).^35^ In combination with our data, these results suggest altered satellite cell dynamics following loss of mechanical stimulation, yet these changes are not necessarily purely increased or decreased content and instead follow non‐linear patterns. In fact, spikes in markers of satellite cell dynamics (such as in the EDL and gastrocnemius) may be a compensatory mechanism to protect muscle mass in these muscle types. Prior works in murine models of muscular dystrophy have found enhanced satellite cell proliferation that coincide with greater muscle size before the onset of myopathies in C57BL6 mice.^36^ As such, it is plausible that robust inductions of these markers in the EDL and gastrocnemius may have conferred some protections to muscle size; conversely, the reduction of these same markers in the soleus may have facilitated the considerable loss in soleus mass. However, the soleus may be more responsive to exercise‐related protections from atrophy. For example, it has recently been hypothesized that oxidative fibres may be more plastic in satellite cell accrual, with MHCI fibres accumulating more satellite cells compared with MHCIIA/X fibres with aerobic exercise training.^37^ Correspondingly, recent research has found resistance training confers greater myonuclei accumulation in soleus muscle compared with the gastrocnemius; more so, myonuclei accrual in the soleus was maintained after 6 months of detraining.^38^ Taken together, the aggregate of evidence implies differential responses to regulators of myogenic activity across muscle types, and these differential responses may correspond to differential susceptibilities to muscle atrophy.

Taken together, our data from across both biological sexes suggest divergent responses to muscle atrophy across different fibre types. Our primary finding is that female mice present with exacerbated catabolic programing and anabolic repression in response to disuse atrophy, which appears to correspond to greater muscle loss compared with male mice. These data provide early evidence of the plausibility sex differences in the aetiology of muscle loss. More works investigating the clinical utility of these differences will be necessary to understand the potential impacts of sex on the aetiology of muscle atrophy. Conversely, it is important to note that this study was predominantly descriptive and more studies investigating mechanisms using in vitro methods will likely be necessary to fully understand how sex interacts with muscle atrophy. Regardless, sex differences may necessitate different clinical interventions for patients undergoing atrophic stimuli. Additionally, our secondary finding was that the same pathology (i.e. disuse) results in dramatically different alterations to cellular signalling depending on the muscle fibre‐type composition. The dissimilarities in response to disuse atrophy across muscle types may partially explain why treatments for muscle atrophies have thus far been unsuccessful and imply that disuse atrophy is more complex than previously thought. Nonetheless, in conclusion, these findings further demonstrate the plausibility for sex differences in muscle pathologies and highlight the importance of investigating muscle pathologies in both sexes in concurrent studies.

## Funding

This study was funded by the National Institutes of Health, Awards R15AR069913/AR/NIAMS and P20GM125503.

## Conflict of interest

None declared.

## Supporting information


**Data S1.** Supporting Information

**Data S2.** Supporting Information

**Figure S1.** Additional presentation of hindlimb and body mass differences with different durations of hindlimb unloading. **A:** Tissue weights normalized to body weight at the initiation of unloading in males. **B:** Tissue weights normalized to body weight at the initiation of unloading in females. **C:** Percent body and tissue weight differences compared to control animals across different durations of unloading in males. **D:** Percent body and tissue weight differences compared to control animals across different durations of unloading in females. Different letters represent statistical differences at Tukey adjusted *p* ≤ 0.05. Female data are italicized and underlined. *n* = 9–12/group.

**Figure S2.** Histograms of frequency distributions for muscle fiber size in males and females across different durations of unloading. **A:** Combined (MHCIIB, MHCX/D, and MHCIIA) fiber distributions in the tibialis anterior of males. **B:** Combined (MHCIIB, MHCX/D, and MHCIIA) fiber distributions in the tibialis anterior of females. **C:** MHCIIB fiber distributions in males. **D:** MHCIIB fiber distributions in females. **E:** MHCIIX/D fiber distributions in males. **F:** MHCIIX/D fiber distributions in females. **G:** MHCIIA fiber distributions in males. H: MHCIIA fiber distributions in females.

**Figure S3.** Fiber type distribution in males and females after different bouts of hindlimb unloading. **A:** Percent of MHCIIB fibers in males and females. **B:** Percent of MHCX/D (unstained) fibers in males and females. **C:** Percent of MHCIIA fibers in males and females. Different letters represent statistical differences at Tukey adjusted *p* ≤ 0.05. *n* = 9‐12/group. *=linear trend within a sex, Ω=quadratic trend within a sex, #=cubic trend within a sex. Female data are italicized and underlined.
