# Supplementary material for: Female mice may have exacerbated catabolic signalling response compared to male mice during development and progression of disuse atrophy
Source: J Cachexia Sarcopenia Muscle. 2021 Mar 5;12(3):717–30. doi: 10.1002/jcsm.12693 (PMC8200438; doi:10.1002/jcsm.12693)
Supplement: Supplementary file 1 — Data S1. Supporting Information [file JCSM-12-717-s005.docx]

**Supplementary Methods**

*Hindlimb unloading*

Hindlimb unloading was performed as we recently described [1]. Briefly, tails of animals were sterilized using ethanol wipes and iodine swab sticks. Tails were then coated with benzoin solution to facilitate tape adhesion. After which, tails were wrapped with athletic tap that was fitted with a loop for the tail suspension apparatus. The tail suspension apparatus was a custom addition to the animal cage, allowing for suspension of the mouse’s tail by a hook and fishing wire attached to the loop on the mouse’s tail. The hook and wire were then attached to a swivel system. This apparatus allowed for animals to move freely around the cage while maintaining unloading. Animals were checked daily to ensure food consumption and general animal welfare.

*Histological Analysis*

Slides were first fixed in ice cold 4% Paraformaldehyde (PFA)/PBS solution for 10 minutes. After 5 minutes wash in PBS, slides were then permeabilized using 0.3% Triton/PBS solution for 10 minutes. After washing with PBS, slides were then blocked with 5% goat serum/PBS solution for 30 minutes (Thermo Fisher, Cat#31873). Slides were then incubated with MHC-IIB primary antibody (Developmental Studies Hybridoma Bank, Cat# BF-F3) diluted 2:25 in 5% goat serum/PBS solution for 2 hours at room temperature. After incubation, slides were washed with PBS, then incubated with FITC fluorescent secondary (Thermo Fisher, Cat#31992) diluted 2:25 in 5% goat serum/PBS solution for 1 hour at room temperature. After secondary incubation, slides were washed with PBS, fixed in 4% PFA/PBS, and washed again in PBS. The same primary and secondary incubation periods were used for MHC-I (Developmental Studies Hybridoma Bank, Cat# BA-F8) using a Rho-Red secondary (Thermo Fisher, Cat# 31660) and MHC-IIA (Developmental Studies Hybridoma Bank, Cat# SC-71) using a DAPI secondary (Abcam, Cat# ab175660). After all primary and secondary incubations were completed, slides were mounted with Vectashield fluorescent mounting media (Vector Laboratories, Cat# H-1000-10). Slides were imaged using FITC, TRITC, and DAPI filters. Muscle fibers were categorized as either MHC-I (red), MHC-IIA (blue), MHC-IIB (green), or MHC-IID/X (absence of stain, but clear presence of fiber). Cross sectional areas for all fiber types were measured to investigate the differential responses between fiber types during disuse atrophy similar to our previous works using succinate dehydrogenase staining [2].

*Fractional Protein Synthesis Rates*

Deuterium labeling of body water was later measured via plasma water-bound hydrogen exchange with a 5% acetone in acetonitrile solution in the presence of 10N sodium hydroxide as described by Yang et al. [3]. Following a twenty-four-hour incubation, acetone was then extracted through the addition of 0.4 mL of hexane and vigorous vortexing. The upper inorganic phase containing acetone in hexane was transferred to a gas-chromatography vial, and were analyzed using an Agilent 7890A Gas Chromatographer (GC) with a HP-5ms capillary column, coupled with an Agilent 5977A Mass Spectrometer (MS). Samples were injected into the GC and separated according to previously published injection, split ratio, and temperature ramping parameters [4, 5]. Plasma water MS analysis was performed in selected ion monitoring (SIM) mode, with acetone being represented by mass-to-charge ratio (m/z) 58 (m+0) and deuterated acetone represented by m/z 59 (m+1), both eluting off of the GC column at approximately 1.5 minutes. Plasma water enrichment was calculated as a ratio of the m+1/m+0 chromatographic peak heights and compared to existing standards of known enrichment.

To measure the deuterium enrichment of protein bound alanine, ~5-20 mg of frozen pulverized mixed muscles (tibialis anterior and gastrocnemius) were homogenized in an ice cold 10% trichloroacetic acid solution, centrifuged to pellet the precipitated proteins as described previously [4-7]. Supernatant was discarded and the protein-rich pellet was washed and disrupted with two additional 10% TCA washes and centrifugation steps to eliminate any potential free amino acids. The protein-rich pellet was then hydrolyzed in 0.2 mL of 6N hydrochloric acid at 105° C for approximately 24 hours. A 50 μl aliquot of each hydrolyzed sample was dried and derivatized with 100 μL of a 3:2:1 solution of N,N-Dimethylformamide dimethyl acetal (Methyl-8), methanol, and acetonitrile, and then transferred to GC vials. Samples were injected into the GC and separated according to previously published injection, split ratio, and temperature ramping parameters [4, 5]. Protein bound alanine MS analysis was performed in SIM mode, with the alanine derivative being represented by m/z 99 (m+0), and the deuterated alanine derivative being represented by m/z 100 (m+1), both eluting off of the GC column at approximately 8.6 minutes. Samples are analyzed following peak integration by applying the corrected area under the curve ratio of m+1/m+0 to a series of enriched alanine standards constructing a secondary linear regression as described by Patterson et al. [8].

The calculation of FSR is accomplished by the following equation:

FSR = E_A_ × [E_BW_ × 3.7 × t] ^−1^ × 100

Where E_A_ represents the enrichment of alanine as measured in the target protein pool, E_BW_ represents the enrichment of body water as measured in the plasma, 3.7 is a constant that accounts for multiple possible deuterium labeling sites on alanine, and t represents the precise time of enrichment from the time of injection to the time of specific muscle excision during harvest. FSR is expressed as the percent of newly synthesized proteins per hour (%·h^-1^).

*Immunoblotting Procedure*

Briefly, powdered gastrocnemius or EDL muscle was homogenized in sample buffer containing 50 mM Tris·HCl, pH 6.8, 1% sodium dodecyl sulfate (SDS), 10% glycerol, 20 mM dithiothreitol, 127 mM 2-mercaptoethanol, and 0.01% bromophenol blue, supplemented with protease inhibitors (Roche, Nutley, NJ) and phosphatase inhibitors (Sigma-Aldrich, St. Louis, MO). Samples were then boiled at 95 ^o^C and centrifuged at 13000 RPM to remove particulates. 20 µg of protein sample was loaded and run on 8-14% SDS polyacrylamide gels, transferred to PVDF membranes, and then blocked for 60 minutes in 5% milk dissolved in tris-buffered saline (TBS). Membranes were then incubated in primary solutions overnight in primary antibody solutions (diluted 1:1000) at 4 ^o^C. After primary incubations, membranes were washed with TBS supplemented with 0.1% Tween-20 (TBST) and incubated for 60 minutes in secondary antibodies (LiCor -IRDye® 800CW or IRDye® 680RD depending on protein of interest and reactivity of the primary antibody). Membranes were finally washed with TBST and imaged using a LiCor Odyssey Fc imaging system (LiCor, Lincoln, NE). All images were normalized to Ponceau S stain and quantified using Image Studio software (LiCor, Lincoln, NE). Primary proteins of interest included: Akt (Cell Signaling, Cat# 9272), pAkt^Ser473^ (Cell Signaling, Cat# 9271), 4EBP1 (Cell Signaling, Cat# 9644), p4EBP1^Thr37/45^ (Cell Signaling, Cat# 2855).

*mRNA Analysis*

Tissues were homogenized in 1 mL of TriZol reagent (Life Technologies, Cat# 15596026, Grand Island, NY ). Afterwards, 200 µL of chloroform was added to samples and samples were vigorously shaken to incorporate chloroform. Samples were then centrifuged at 20,000g for 25 minutes at 4 ^o^C. mRNA was then isolated using a commercial kit (Ambion Purelink RNA mini kit, Cat# 12183020, Life Technologies). 1 µg of mRNA was then reverse transcribed into cDNA using commercial reagents (Superscript Vilo, Cat#11755500, Life Technologies). cDNA was then diluted to 1:100 and analyzed for genes of interest. mRNA analysis of genes related to protein synthesis and degradation were measured as we have previously described using SYBR primers or Taqman probes as appropriate [9]. Genes of interest included: *18s* (Clone #Mm03928990_g1), *Redd1* (Clone #Mm00512504_g1), *Deptor* (Clone #Mm01195339_m1), *Atrogin* (Clone #Mm00499523_m1), *Murf1* (Clone #Mm01185221_m1), Gadd45a (Clone # Mm00432802_m1), *Ubc* (Clone #Mm02525934_g1), *Pax7* (Clone #Mm01354484_m1), *Igf1* (Clone #Mm00439560_m1), *MyoG* (Clone #Mm00446194_m1), *MyoD* (Clone #Mm00440387_m1), *Mki67* (Clone #Mm01278617_m1), and *Ccnd1* (Clone #Mm00432359_m1). SYBR primers for Pgc1α4 have been previously reported [10]. 18s was not different between groups. All samples were analyzed using a ThermoFisher StepOne System (ThermoFisher, Waltham, MA).

References

1. ME R-C, S L, WS H, LT J, LC W, MG A, et al. Altering aspects of mitochondrial quality to improve musculoskeletal outcomes in disuse atrophy. Journal of applied physiology (Bethesda, Md : 1985). 2020;doi:10.1152/japplphysiol.00407.2020

2. Brown JL, Rosa-Caldwell ME, Lee DE, Blackwell TA, Brown LA, Perry RA, et al. Mitochondrial degeneration precedes the development of muscle atrophy in progression of cancer cachexia in tumour-bearing mice. J Cachexia Sarcopenia Muscle. 2017;8:926-38. doi:10.1002/jcsm.12232

3. Yang D, Diraison F, Beylot M, Brunengraber DZ, Samols MA, Anderson VE, et al. Assay of low deuterium enrichment of water by isotopic exchange with [U-13C3] acetone and gas chromatography–mass spectrometry. Analytical biochemistry. 1998;258:315-21.

4. Nilsson MI, Greene NP, Dobson JP, Wiggs MP, Gasier HG, Macias BR, et al. Insulin resistance syndrome blunts the mitochondrial anabolic response following resistance exercise. American Journal of Physiology-Endocrinology And Metabolism. 2010;299:E466-E74.

5. Gasier HG, Riechman SE, Wiggs MP, Previs SF, Fluckey JD. A comparison of 2H2O and phenylalanine flooding dose to investigate muscle protein synthesis with acute exercise in rats. American Journal of Physiology - Endocrinology and Metabolism. 2009;297:E252-E9. doi:10.1152/ajpendo.90872.2008

6. Lee DE, Brown JL, Rosa ME, Brown LA, Perry RA, Wiggs MP, et al. microRNA‐16 Is Downregulated During Insulin Resistance and Controls Skeletal Muscle Protein Accretion. Journal of Cellular Biochemistry. 2016;117:1775-87. doi:doi:10.1002/jcb.25476

7. Shimkus KL, Shirazi-Fard Y, Wiggs MP, Ullah ST, Pohlenz C, Gatlin DM, 3rd, et al. RESPONSES OF SKELETAL MUSCLE SIZE AND ANABOLISM ARE REPRODUCIBLE WITH MULTIPLE PERIODS OF UNLOADING/RELOADING. Journal of applied physiology (Bethesda, Md : 1985). 2018;doi:10.1152/japplphysiol.00736.2017

8. Patterson BW, Zhao G, Klein S. Improved accuracy and precision of gas chromatography/mass spectrometry measurements for metabolic tracers. Metabolism. 1998;47:706-12.

9. Greene NP, Lee DE, Brown JL, Rosa ME, Brown LA, Perry RAJ, et al. Mitochondrial quality control, driven by PGC-1α, is dysregulated by Western Diet-induced obesity and partially restored by moderate physical activity in mice. Physiological Reports. 2015;3:e12470. doi:10.14814/phy2.12470

10. Brown JL, Rosa-Caldwell ME, Lee DE, Brown LA, Perry RA, Shimkus KL, et al. PGC-1alpha4 gene expression is suppressed by the IL-6-MEK-ERK 1/2 MAPK signalling axis and altered by resistance exercise, obesity and muscle injury. Acta Physiol (Oxf). 2017;220:275-88. doi:10.1111/apha.12826
