# Supplementary material for: Female mice may have exacerbated catabolic signalling response compared to male mice during development and progression of disuse atrophy
Source: J Cachexia Sarcopenia Muscle. 2021 Mar 5;12(3):717–30. doi: 10.1002/jcsm.12693 (PMC8200438; doi:10.1002/jcsm.12693)
Supplement: Supplementary file 2 — Data S2. Supporting Information [file JCSM-12-717-s002.docx]

**Supplementary Results**

*Muscle Weight Data*

Males had lower bodyweight with increased duration of unloading, with a linear decrease trend (p=0.010, Table 1) observed in body weight across groups. Females had a quadratic trend for body weight (p=0.010, Table 1), with a lower body weights in animals with longer durations of unloading that appeared to plateau at 72 hr of disuse. In male mice, gastrocnemius muscle exhibited a linear trend, with increased duration of hindlimb unloading corresponding to lower mass (p<0.001, Table 1), 48 hr and 168 hr animals had ~12.1% and ~15.8% lower gastrocnemius weight compared to 0hr. Similarly, in female gastrocnemius muscle, a linear trend was also noted (p<0.001, Table 1). With 48 hr, 72 hr, and 168 hr female animals having ~ 8.7%, ~15.0%, and ~16.9% lower gastrocnemius weights compared to 0 hr. In male soleus muscle, a linear trend was found (p<0.001, Table 1), with 48 hr, 72 hr and 168 hr animals having ~14.0%, ~19.6%, and ~38.6% lower soleus muscle weights compared to 0 hr. In females, a linear trend was also found (p<0.001, Table 1), with 24 hr, 48 hr, 72 hr, and 168 hr animals having ~10.9%, ~17.9%, ~24.0%, and ~33.8% lower soleus muscle weights compared to 0 hr females. In male plantaris muscle, a linear trend was noted (p<0.001, Table 1), with 48 hr, 72 hr, and 168 hr mice having ~11.1%, ~11.2%, and ~18.0% lower plantaris weights compared to 0 hr. Likewise, female mice demonstrated a linear trend (p<0.001, Table 1), with 24 hr, 48 hr, 72 hr, and 168hr animals having ~15.7%, ~16.5%, ~25.1%, and ~20.2% lower plantaris weights compared to 0 hr animals. In male tibialis anterior (TA) muscle, a linear trend was found with duration of hindlimb unloading (p<0.001, Table 1), with 168 hr animals having ~13.4% lower TA weights compared to 0 hr. In female TA muscle, a linear trend was also noted (p=0.008, Table 1) with 48 hr, 72 hr and 168 hr animals having ~9.1%, ~14.7%, and 11.8% lower TA weights compared to 0 hr. However, in both male and female mice there was no effect of hindlimb unloading on EDL weights (p=0.334 and p=0.203 respectively).

*Histology Results*

Within the TA muscle, across all fiber types, males had a significant quadratic trend in average area of all fibers (p=0.0008), with 72 hr and 168 hr animals having ~32% and ~21% smaller average fiber areas compared to 0 hr animals (Figure 1B & 1F). Females had a significant quadratic trend in average area of all fibers (p=0.0001), with 48 hr, 72 hr, and 168 hr animals having ~29%, ~40%, and ~34% smaller average fiber area compared to 0 hr animals (Figure 1B & 1F). In males, there was no significant pairwise differences or trends noted in MHCIIB fiber area across any groups (p=0.06-0.18, Figure 1C & 1F). However, females had a significant quadratic trend in MHCIIB fibers (p=0.0001), with 48 hr, 72 hr and 168 hr animals having ~36%, ~41%, and ~33% smaller fiber areas compared to 0 hr (Figure 1C & 1F). Males had a significant quadratic trend in non-stained (MHCX/D) fibers (p=0.002), with 48 hr, 72 hr, and 168 hr animals having ~28%, 38%, and 40% smaller areas compared to 0 hr animals (Figure 1D & 1F). Similarly, females had a significant quadratic trend in non-stained (MHCX/D) fibers (p=0.003), with 48 hr, 72 hr and 168 hr animals having ~26%, 35%, and 36% smaller areas compared to 0 hr animals (Figure 1D & 1F). Males had a significant quadratic trend in MHCIIA fibers (p=0.0002), with 24 hr, 48 hr, 72 hr, 168 hr animals having ~26%, ~34%, ~40% and 35% smaller fiber areas compared to 0 hr (Figure 1E & 1F). Likewise, females had a significant quadratic trend in MHCIIA fibers (p=0.012), with 24 hr, 72 hr, and 168 hr animals having ~29%, 31%, and 29% smaller fiber area compared to 0 hr (Figure 1E & 1F). The difference between 48 hr animals and 0 hrs was not statistically significant; however there was a mean difference for 48 hr animals to have ~18% smaller fiber area compared to 0 hr (Figure 1E & 1F).
