# Supplementary material for: Female mice may have exacerbated catabolic signalling response compared to male mice during development and progression of disuse atrophy
Source: J Cachexia Sarcopenia Muscle. 2021 Mar 5;12(3):717–30. doi: 10.1002/jcsm.12693 (PMC8200438; doi:10.1002/jcsm.12693)
Supplement: Supplementary file 3 — Figure S1. Additional presentation of hindlimb and body mass differences with different durations of hindlimb unloading. A: Tissue weights normalized to body weight at the initiation of unloading in males. B: Tissue weights normalized to body weight at the initiation of unloading in females. C: Percent body and tissue weight differences compared to control animals across different durations of unloading in males. D: Percent body and tissue weight differences compared to control animals across different durations of unloading in females. Different letters represent statistical differences at Tukey adjusted p ≤ 0.05. Female data are italicized and underlined. n = 9–12/group. [file JCSM-12-717-s001.pdf]

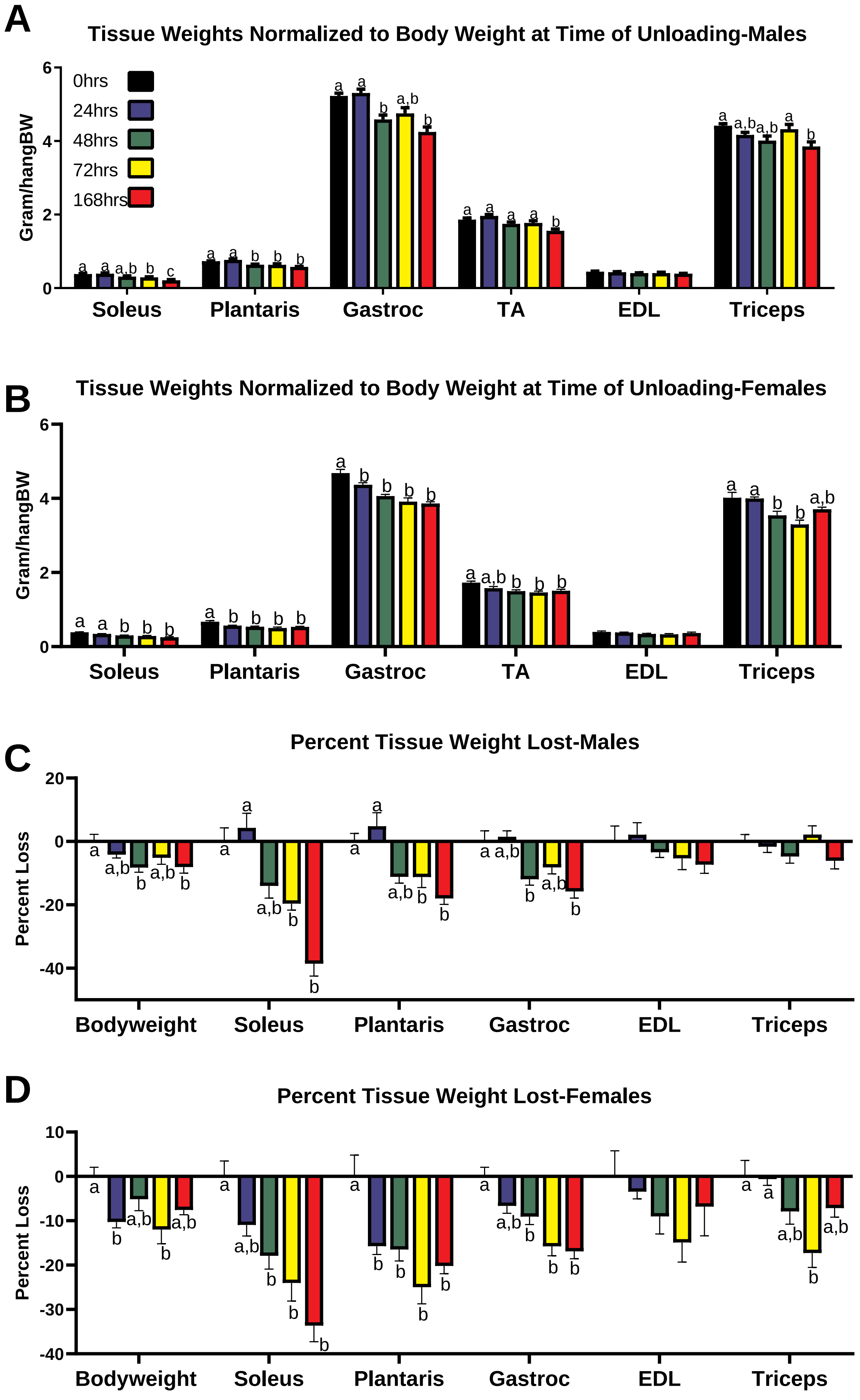
A
Tissue Weights Normalized to Body Weight at Time of Unloading-Males
Gram/hangBW
0hrs
24hrs
48hrs
72hrs
168hrs
Soleus
Plantaris
Gastroc
TA
EDL
Triceps
B
Tissue Weights Normalized to Body Weight at Time of Unloading-Females
Gram/hangBW
Soleus
Plantaris
Gastroc
TA
EDL
Triceps
C
Percent Tissue Weight Lost-Males
Percent Loss
Bodyweight
Soleus
Plantaris
Gastroc
EDL
Triceps
D
Percent Tissue Weight Lost-Females
Percent Loss
Bodyweight
Soleus
Plantaris
Gastroc
EDL
Triceps
