# Supplementary figures and images for: Female mice may have exacerbated catabolic signalling response compared to male mice during development and progression of disuse atrophy
Source: J Cachexia Sarcopenia Muscle. 2021 Mar 5;12(3):717–30. doi: 10.1002/jcsm.12693 (PMC8200438; doi:10.1002/jcsm.12693)

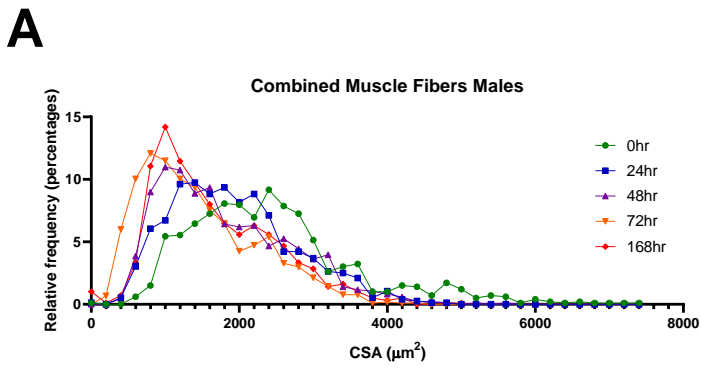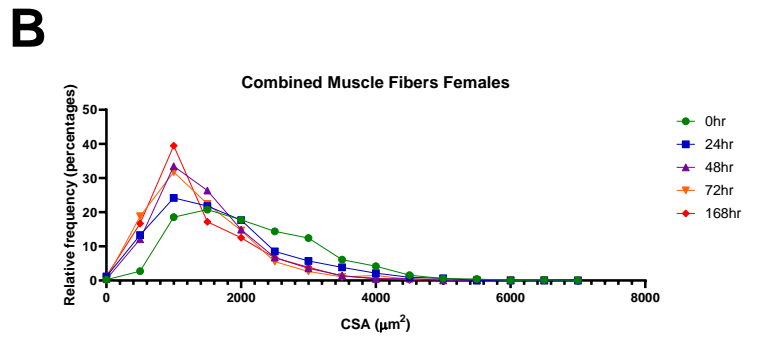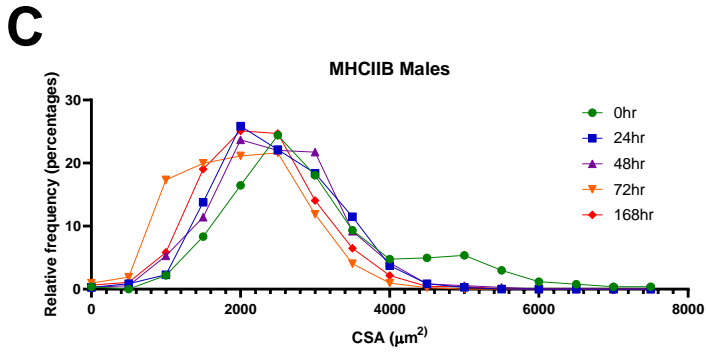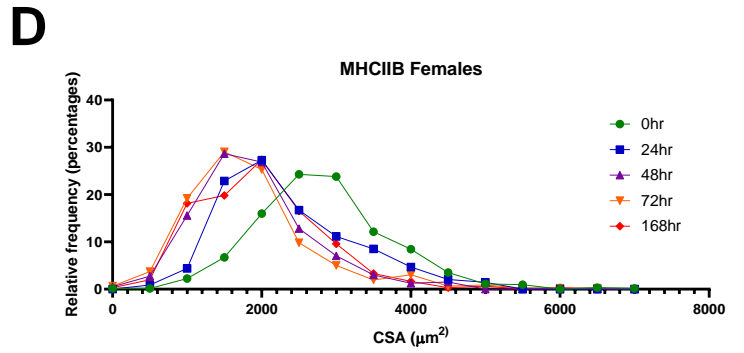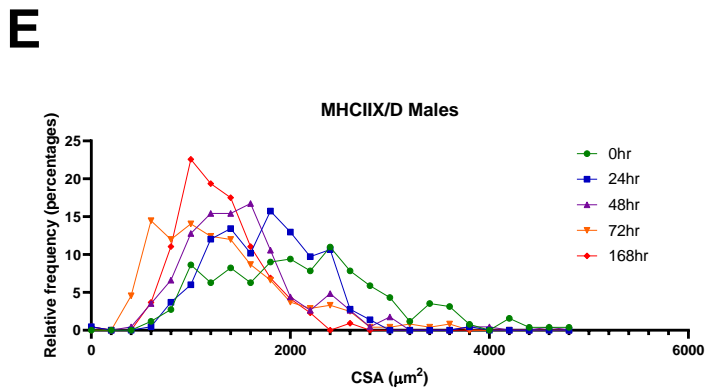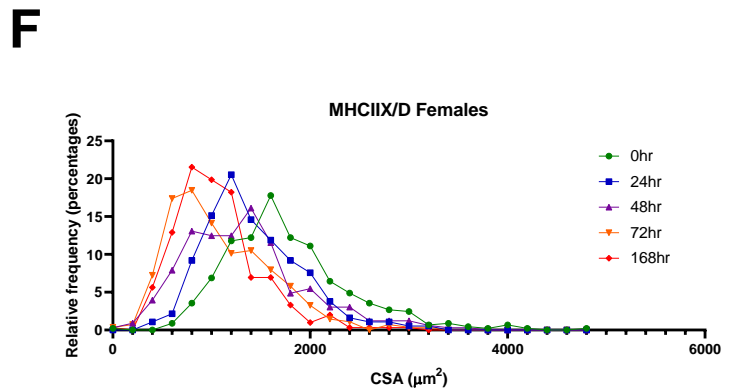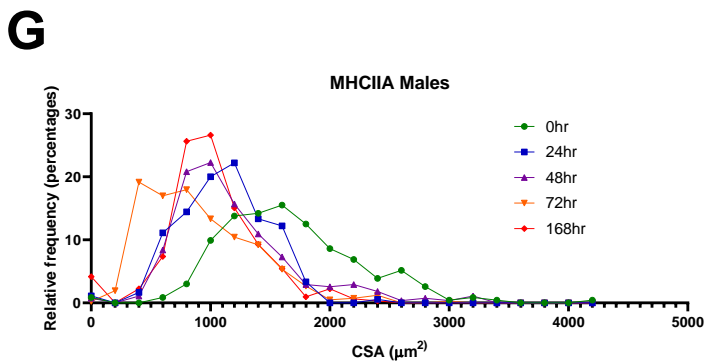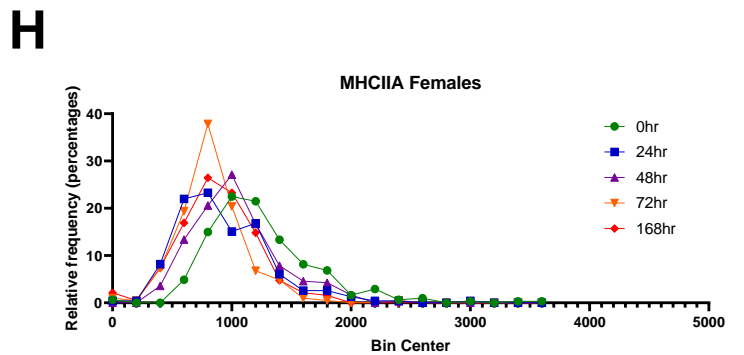

Supplement: Supplementary file 4 — Figure S2. Histograms of frequency distributions for muscle fiber size in males and females across different durations of unloading. A: Combined (MHCIIB, MHCX/D, and MHCIIA) fiber distributions in the tibialis anterior of males. B: Combined (MHCIIB, MHCX/D, and MHCIIA) fiber distributions in the tibialis anterior of females. C: MHCIIB fiber distributions in males. D: MHCIIB fiber distributions in females. E: MHCIIX/D fiber distributions in males. F: MHCIIX/D fiber distributions in females. G: MHCIIA fiber distributions in males. H: MHCIIA fiber distributions in females. [file JCSM-12-717-s004.pdf]

**A**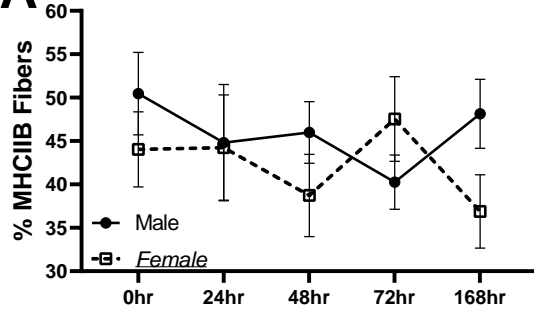**B**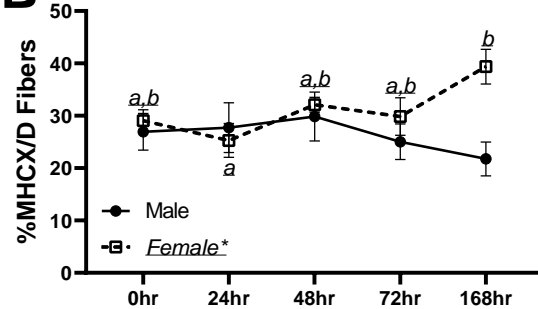**C**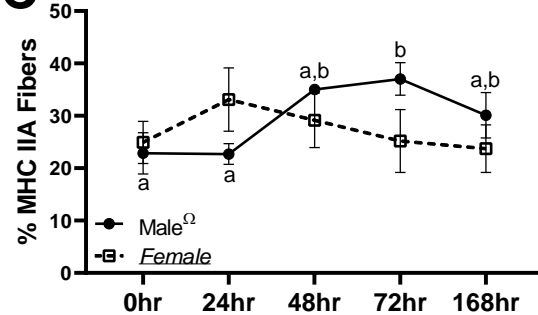

Supplement: Supplementary file 5 — Figure S3. Fiber type distribution in males and females after different bouts of hindlimb unloading. A: Percent of MHCIIB fibers in males and females. B: Percent of MHCX/D (unstained) fibers in males and females. C: Percent of MHCIIA fibers in males and females. Different letters represent statistical differences at Tukey adjusted p ≤ 0.05. n = 9‐12/group. *=linear trend within a sex, Ω=quadratic trend within a sex, #=cubic trend within a sex. Female data are italicized and underlined. [file JCSM-12-717-s003.pdf]
